# Chemical Composition, Larvicidal and Repellent Activities of Wild Plant Essential Oils against *Aedes aegypti*

**DOI:** 10.3390/biology12010008

**Published:** 2022-12-21

**Authors:** Muhammad Ghazanfar Abbas, Abdullah Haris, Muhammad Binyameen, Abdul Nazir, Raimondas Mozūratis, Muhammad Azeem

**Affiliations:** 1 Laboratory of Insect Chemical Ecology, Department of Entomology, Faculty of Agricultural Sciences & Technology, Bahauddin Zakariya University, Multan 60800, Pakistan; 2 Department of Environmental Sciences, Abbottabad Campus, COMSATS University Islamabad, Abbottabad 22060, Pakistan; 3 Department of Zoology, Stockholm University, 10691 Stockholm, Sweden; 4 Laboratory of Chemical and Behavioural Ecology, Institute of Ecology, Nature Research Centre, LT-08412 Vilnius, Lithuania; 5 Department of Chemistry, Abbottabad Campus, COMSATS University Islamabad, Abbottabad 22060, Pakistan

**Keywords:** eco-friendly, essential oils, gas chromatography-mass spectrometry, mosquitoes, toxicity, bioactive compounds

## Abstract

**Simple Summary:**

Mosquitoes are the deadliest insects alive due to the transmission of pathogens that cause diseases. Plant essential oils are considered an alternative to synthetic repellents for controlling mosquitoes. We have investigated the repellent and larvicidal activity of six plant essential oils against adult female mosquitos and the larvae of yellow fever mosquitos, *Aedes aegypti*. The essential oils extracted from *Mentha longifolia*, *Zanthoxylum armatum*, *Erigeron bonariensis*, and *E. canadensis* have the potential to manage *Ae. aegypti* at the larval stage. Moreover, *M. longifolia*, *E. canadensis*, *E. bonariensis*, and *Salsola imbricata* essential oils exhibited prolonged mosquito-repellent activity against adult female *Ae. Aegypti;* these oils might be used to develop formulations that are efficient and cost-effective as mosquito repellents without harming humans and the environment.

**Abstract:**

Bio-degradable and eco-friendly essential oils (EOs) extracted from *Mentha longifolia*, *Salsola imbricata*, *Erigeron bonariensis*, *E. canadensis*, *Ailanthus altissima*, and *Zanthoxylum armatum* were investigated for their repellent and larvicidal potential against *Aedes aegypti mosquitoes*. The EOs of *M. longifolia*, *S. imbricata*, *E. bonariensis*, *E. canadensis*, *A. altissima*, and *Z. armatum* exhibited 99.0%, 96.8%, 40.2%, 41.7%, 29.1%, and 13.2% repellency against *mosquitoes* at a tested dose of 33.3 μg/cm^2^, respectively. In time span bioassays, the EOs of *M. longifolia*, *S. imbricata*, *E. bonariensis*, and *E. canadensis* showed more than 40% repellency for 60 min at a tested dose of 330 μg/cm^2^. Larvicidal bioassays revealed that larvae of *Ae. aegypti* were the most susceptible to *M. longifolia* (LC_50_, 39.3 mg/L), *E. bonariensis* (LC_50_, 26.0 mg/L), *E. canadensis* (LC_50_, 35.7 mg/L), and *Z. armatum* (LC_50_, 35.9 mg/L) EOs upon 48 h exposure. The most abundant constituents in the EOs of *M. longifolia*, *S. imbricata*, *E. bonariensis*, *E. canadensis* and *A. altissima* were piperitone oxide (45.5%), carvone (39.9%), matricaria ester (43.1%), (31.7%) and eugenol (24.4%), respectively. Our study demonstrates that EOs of *M. longifolia*, *S. imbricata*, *E. bonariensis*, and *E. canadensis* might be used to control *Ae. aegypti* mosquitoes without harming humans or the environment.

## 1. Introduction

Mosquitoes are the deadliest insects [1] due to the transmission of pathogens, causing diseases such as the West Nile virus, filariasis, dengue, chikungunya, Japanese encephalitis, and malaria in humans [2]. The yellow fever mosquito, *Aedes aegypti* L. (Diptera: Culicidae) is a primary vector of the Zika virus, chikungunya, yellow fever, and dengue viruses [2]. Moreover, it is listed as a major vector threat in the world due to its proliferation ability [3]. In the last few decades, the incidence of diseases spread by *Ae. aegypti* increased all around the world. In 1969, the epidemic of dengue was present in 9 countries, but now it has spread to more than 100 countries. The incidence of dengue has increased dramatically, and about half the population of the world is at risk of contracting this virus from the yellow fever mosquito [4]. Asia suffers more than 70% of the global burden of diseases due to dengue mosquitoes [2], and the people of Pakistan are at risk due to dengue. Dengue infections in Pakistan are mostly reported in September and October [5,6]. Dengue is considered a dangerous disease due to difficulty in control and diagnosis [7]. Therefore, one of the means to battle this disease is a reduction in *Ae. aegypti* populations.

Synthetic insecticides like deltamethrin, temephos, acetamiprid, metofluthrin, and cypermethrin have been found to be effective against *Ae. aegypti* [8,9,10,11]. However, resistance in *Ae. aegypti* has also been reported against permethrin, deltamethrin, and temephos [12,13,14,15]. Chemical insecticides are a danger to non-target organisms [16,17] causing endocrine, reproductive, and carcinogenic problems in humans [18,19]. *N*, *N*’-diethyl-3-methylbenzamide (DEET) is a common mosquito repellent [20]. However, the extensive use of DEET has also resulted in harmful effects like allergic reactions and skin irritation, and is also responsible for causing brain disease—encephalopathy—in children [21,22]. Keeping in mind the problems associated with chemical insecticides, synthetic repellents, and diseases spread by *Ae. aegypti*, there is a need to find natural chemical sources to develop new plant-based mosquito repellents and insecticides.

Plants-based products are not only safe to use but are also not harmful to humans and animals [23]. Furthermore, plant-based materials have proven very effective against blood-seeking insects. For example, some components of plant-based essential oils like safrole, myristicin, terpinolene, and α-terpineol showed higher efficiency against these insects than DEET [24]. Some other essential oils have also proven to be very effective in killing and repelling the *Aedes* species [19,25,26]. Plant essential oils are considered an alternative for controlling insect pests. In the market, plant-based chemicals represent an estimated USD seven hundred million and forty-five thousand tons of the total world pesticide production. Furthermore, due to their degradability, these are safe to use for humans and the environment [27].

The present study reports the repellent and larvicidal activity of six plant essential oils (EOs), derived from *Mentha longifolia*, *Salsola imbricata*, *Erigeron bonariensis*, *E. canadensis*, *Ailanthus altissima*, and *Zanthoxylum armatum*, against adult females and larvae of *Ae. aegypti*.

## 2. Materials and Methods

### 2.1. Collection of Plant Material

Leaves and stems of *M. longifolia*, *S. imbricata*, *E. bonariensis*, *E. canadensis*, *A. altissima,* and *Z. armatum* were collected from the Bio-Park at Bahauddin Zakaria University, Multan and from a hilly area of Abbottabad, Pakistan during September–October. The species of the plants were identified by a plant taxonomist at COMSATS University Islamabad, Abbottabad Campus, Abbottabad, Pakistan as well as using the Plant-Net Identifier Software, Version 2.0, (Sydney, Australia).

### 2.2. Extraction of Essential Oils

The steam distillation method was used to extract essential oils from fresh parts of identified plants on the same day as their collection. The steam distillation method is described in detail by Azeem et al. [28,29]. Briefly, about 2 kg of fresh plant biomass was cut into small pieces and placed into the distillation apparatus. Two litres of distilled water were added to the bottom of the distillation apparatus and plant material was loaded above the water level. The distillery was heated by using an electric heating mantle. The distillate, comprising water and plant volatiles, was collected in a separating funnel for 4 h. The distillate was extracted in HPLC-grade n-hexane (70 mL × 3). The extracted organic layer was dried by the addition of anhydrous MgSO_4_. After filtration, the solvent was evaporated with a low-pressure rotary evaporator at 25 °C. The mass of extracted essential oil was compared with the mass of fresh plant materials to calculate the percentage yield of extracted essential oil. The essential oil was stored in screw-capped glass vials at −20 °C until use for chemical analysis and bioassays.

### 2.3. Rearing of Ae. aegypti

The *Ae. aegypti* colony was reared in the laboratory, using the method described by Johnson [30] and Zheng et al. [31]. The larval population of *Ae. aegypti* was taken from Health Department, Multan, Pakistan. Larvae were placed in a plastic container (20 × 16 × 4 cm) filled with water. A fish diet (Osaka green fish food) consisting of 3% crude fat, 4% crude fibre, and 28% crude protein was used for larval feeding. The pupae collected daily from the larval container were transferred to plastic cups (350 mL capacity) filled with distilled water (200 mL). They were then placed in a separate Plexiglass cage (30 × 30 × 30 cm), with 3 meshes (one on the upper side and one on each lateral side) as well as an opening hole (18 cm diameter) covered with a muslin cloth fastened by a rubber band. Cotton soaked with a 10% sugar solution was kept in the cages as a food for adults. *Ae. aegypti* were used to mate after 3–4 days of emergence, and therefore the adult females were fed on the blood of constrained pigeons. The plastic cups (350 mL capacity), lined with 10 cm long wax paper and filled with 120 mL of water, were placed in the adult cage of blood-fed female mosquitoes for oviposition. Egg laying was observed after 3–5 days of mating. The eggs laid by the female mosquitoes were either placed in a larval container filled with water for hatching or stored in a dry place for whenever eggs may be needed for use [32]. Only mated females and larvae of *Ae. aegypti* were used in the repellent and larvicidal bioassays, respectively. Rearing as well as experiments were performed in a climatic chamber where the relative humidity was 70 ± 10%, the temperature was 28 ± 2 °C, and a photoperiod of 14:10 h light: dark was maintained.

### 2.4. Mosquito Repellency Bioassay

The repellency of extracted essential oils was investigated by using the human bait method against female *Ae. aegypti* before the scotophase period. The essential oils solutions at concentrations of 1, 5, and 10% were prepared in ethanol to evaluate their repellency against adult female mosquitoes. Thirty mated and blood-starved four-to-five day-old female mosquitoes were separated in an adult cage (30 × 30 × 30 cm) 24 h before the repellency experiment. Before the start of the bioassay, the hands of the subject were washed with scent-free soap and then dried. Afterwards, gloves were worn on the hands to cover the entire hands, except for a circular area of 30 cm^2^ on the dorsal side of the hands. A 100 μL aliquot of solvent or solution of the test substance was applied on the exposed area of the hand in each replication of treatments, giving doses of 33.3 µg/cm^2^, 166.5 µg/cm^2^, and 330 µg/cm^2^ when 1%, 5%, and 10% concentrations of EO were used. After 3 min drying at room temperature, the solvent or substance-treated hands were exposed to female *Ae. aegypti* in the experimental units for five min. The number of female *Ae. aegypti* landings on the negative control and test-treated exposed area of hands were counted. The human subjects (volunteers) were informed about the test procedure and consent was obtained before conducting repellency bioassays, moreover, permission regarding human subject use was also obtained from the Ethical and Biosafety Committee of Bahauddin Zakariya University. The percentage repellency was calculated by adopting the formula reported by Azeem et al. [29]: percentage repellency = [(M_c_ − M_t_)/M_c_] × 100, where M_c_ is the number of mosquito landings on the negative control (solvent) treated hand and M_t_ is the number of mosquito landings on the test substance treated hand. The essential oils that showed more than 60% repellency against *Ae. aegypti* at each tested dose and were further investigated to evaluate the time span repellency (repellent longevity). The time span bioassay was carried out in the same way as the repellency bioassay described above, except this time using the same treated hand after each 15 min period and counting females landing for 5 min until the number of mosquito landings on the control and treated hands became equal. All the repellency bioassays were repeated five times, randomly, to minimize the error in the experiment.

### 2.5. Larvicidal Activity Bioassay

The larvicidal activity of selected essential oils was tested against the second-instar larvae of *Ae. aegypti* by following the protocol explained by Ali et al. [33], with some modifications. Briefly, five second-instar larvae of *Ae. aegypti* were added to each portion of the ice tray (50 mL capacity), which had 20 mL water. The larvicidal activity of the essential oils was evaluated at different concentrations where it remained effective. Different dilutions of essential oils were prepared in DMSO, and 50 µL essential oil solution or DMSO was added to each well. Thus, the final concentration of essential oil in wells was 6.25 mg/L to 1600 mg/L (6.25, 12.5, 25, 50, 100, 200, 400, 800, 1600 mg/L) with twofold dilution at each step. DMSO was used as the negative control and its concentration in test media never exceed 0.25%. The larvae were exposed to essential oils or the negative control for 24 and 48 h to evaluate susceptibility. A fish diet was also provided to larvae during the exposure period. After the exposure period, larvae mortality was checked by using a camel hairbrush, and the larvae that did not show any movement were considered dead. At least seven replicates of each concentration of different essential oils and control were employed.

### 2.6. Chemical Analysis of the Essential Oils

Essential oils that showed good repellency against *Ae. aegypti* were investigated further, using a Hewlett Packard gas chromatography-mass spectrometry (GC–MS) system (Agilent Technologies Inc., Santa Clara, CA, USA), to detect the main components of the essential oils. The 6890N GC was equipped with a 30 m long capillary column with 0.25 mm internal diameter and 0.25 μm stationary phase film thickness. The stationary phase of the GC column was 95% dimethylpolysiloxane and 5% diphenyl (DB-5, Agilent Technologies Inc., Santa Clara, CA, USA). The injector of GC was constantly operated at a temperature of 235 °C. The temperature of the GC oven was set as follows: initially, it was kept constant at 40 °C for 2 min, then raised to 240 °C at a constant rate of 4 °C/min and finally was programmed isothermally at 240 °C for 8 min. Helium (gas) was used as a mobile phase at a constant flow of 1 mL/min. An aliquot of 1 µL dilute solution of essential oil was injected into GC in the splitless mode for 30 s. The mass spectrometer parameters were programmed as follows: electron energy for ionization was maintained at 70 eV, ion source temperature was set at 180 °C, and the range of mass spectra scan was set at 30–400 amu. To calculate the composition (%) of compounds in essential oils, a total ion chromatogram was used. Separated compounds were initially identified through the comparison of mass spectra with the NIST-2008 MS library. In addition, the retention indices of separated compounds were compared to published data and the NIST online library. To calculate retention indices of separated compounds, the standard mixture of n-alkanes (C_9_–C_24_) was analyzed using the same GC-MS parameters as were used for essential oils. A final verification of the compounds was carried out by injecting the solution of pure available standard compounds at the same conditions used for essential oils analysis. The standard compounds were purchased from Sigma-Aldrich (St. Louis, MI, USA) or Alfa Aesar (Haverhill, MA, USA) chemical suppliers, or otherwise purified in a laboratory at the same parameters used for essential oils analysis.

### 2.7. Statistical Analysis

General Linear Model (GLM) was used to evaluate the EO type and dose effect on the repellency of mosquitoes in repellency bioassay as well as EO type and time effect on the repellency in time span experiments. In all models, experimental replication was treated as a random variable. If a significant effect was determined, pairwise comparisons of group means by Tukey’s *post hoc* test at the significance threshold (alpha = 0.05) were used. The statistical analysis was performed by Statistica software version 14.0.1.25 (TIBCO Software Inc, Palo Alto, CA, USA). For larvae mortality data, the Abbott formula [34] was used to calculate corrected mortality. The different lethal concentrations LC_50_ and LC_90_ were calculated by using probit analysis through the Polo-Plus software. The LC_50_ values of the two bioassays were considered significantly different when their fiducial limits did not overlap [35].

## 3. Results

### 3.1. Yield of Essential Oils

The *Z. armatum* leaves were the richest in essential oil and yielded 0.76%, whereas the least amount of essential oil was obtained from *S. imbricata* and *A. altissima*, which produced yields of 0.01% and 0.04%, respectively (Table 1).

### 3.2. Repellency of Essential Oils

**Figure 1:**
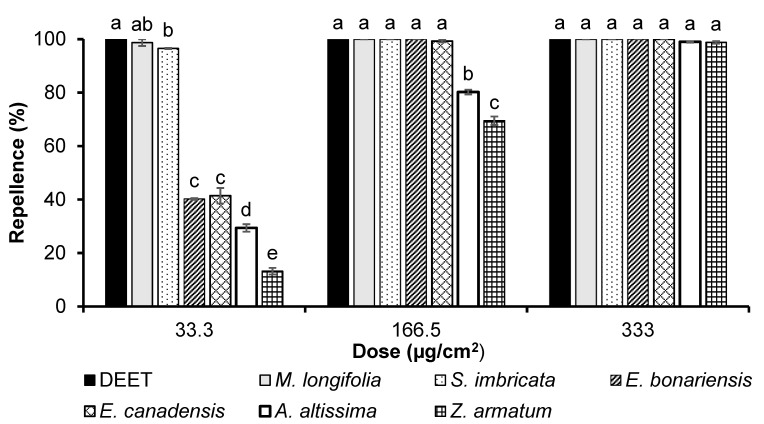
Repellency exhibited by DEET and essential oils, tested at the doses of 33.3 µg/cm^2^, 166.5 µg/cm^2^, and 330 µg/cm^2^ against *Ae. aegypti female*. Bars, indicated by different letters, denote significant differences (*p *< 0.05) among different treatments when tested at the same concentration by ANOVA post hoc Tukey test. Error bars denote the standard error (n = 5).

A statistical data evaluation by GLM revealed that the EO type (*df *= 6, *F* = 1178, *p *< 0.001) and dose (*df *= 2, *F* = 5423, *p *< 0.001) significantly affected the repellency of mosquitoes. Pairwise comparisons of group means by Tukey’s post hoc test at the significance threshold (*alpha* = 0.05) revealed that the essential oil of *M. longifolia* showed high repellent activities, comparable to those of DEET, at the lowest dose of 33.3 µg/cm^2^ (Figure 1). EO of *S. imbricate* had a good repellent effect as well, while essential oils of *E. bonariensis*, *E. canadensis*, and *A. altissima* demonstrated from 30% to 40% of DEET efficiency. The essential oil of *Z. armatum* showed the lowest repellency compared to all tested essential oils. At the medium dose of 166.5 µg/cm^2^, the essential oils of *A. altissima* and *Z. armatum* were significantly weaker repellents compared to the rest of the samples, whose activities did not differ significantly from each other. The repellent activity of six essential oils did not differ significantly from DEET tested directly after application at a dose of 330 µg/cm^2^ concentration (Figure 1).

#### 3.2.1. Time Span Repellency at a Dose of 33.3 µg/cm^2^

**Figure 2:**
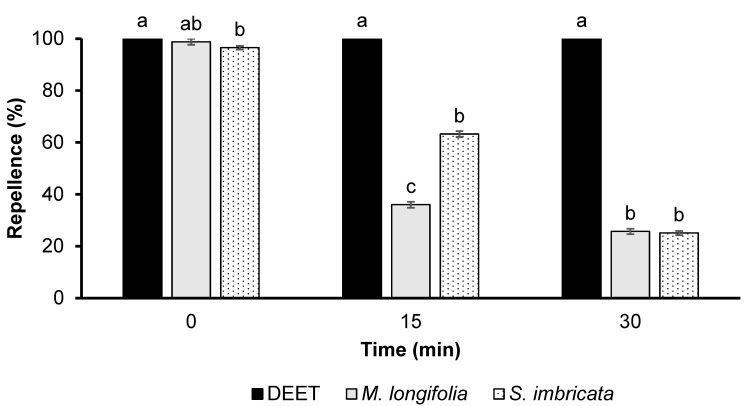
Time-based repellency of DEET and two essential oils tested at a dose of 33.3 µg/cm^2^ against *Ae. aegypti*. Bars having different letters depict significant differences (*p *< 0.05) among the repellency of different tested substances at 0, 15, and 30 min of post-treatment when compared independently by ANOVA post hoc Tukey test. Error bars denote the standard error (n = 5).

The GLM model showed significant effects of EO type (*df* = 2, *F* = 2970, *p *< 0.001) and time (*df* = 2, *F* = 4239, *p *< 0.001) on the repellency effect. EO of *M. longifolia* and *S. imbricata* showed repellency comparable to DEET immediately after application. However, their repellency decreased drastically after 15 and 30 min of application (Figure 2). *S. imbricata* exhibited 63% repellency after 15 min, which was significantly higher than the repellency of *M. longifolia*. However, after 30 min, both plants’ essential oils exhibited similar activity (Figure 2).

#### 3.2.2. Time Span Repellency at a Dose of 166.5 µg/cm^2^

**Figure 3:**
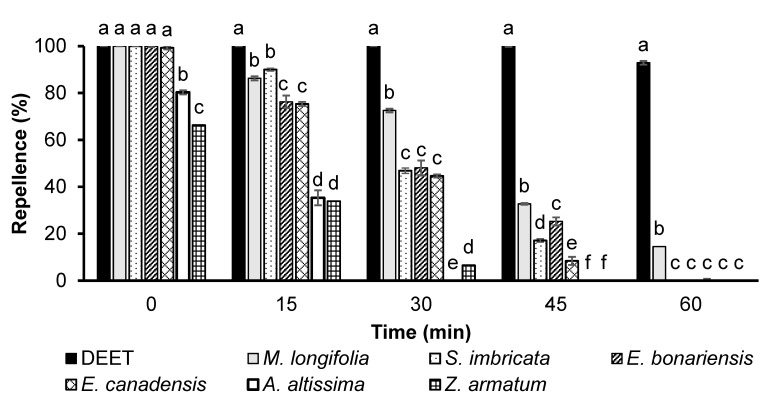
Time-based repellency of DEET and essential oils at a dose of 166.5 µg/cm^2^ against *Ae. aegypti*. Bars having different letters depict significant differences (*p* < 0.05) among the repellency of different tested substances at different periods of post-treatment that were compared for each period independently by ANOVA post hoc Tukey test. Error bars denote the standard error (n = 5).

GLM analyses revealed significant effects of EO type (*df* = 4, *F* = 8625, *p *< 0.001) and time (*df* = 4, *F* = 8559, *p *< 0.001) on the repellency effect. Four out of six plants’ essential oils exhibited 100% repellency at a dose of 166.5 µg/cm^2^ when tested immediately after application. However, their repellencies decreased when tested after 15 or 30 min. *A. altissima* and *Z. armatum* exhibited significantly lower repellency compared to all other essential oils or the positive control (Figure 3). The essential oil of *M. longifolia* proved best in repellency at a dose of 166.5 µg/cm^2^ and showed 33% repellency after 45 min of treatment, which was significantly higher than that of all other essential oils, but significantly lower than that of DEET (Figure 3).

#### 3.2.3. Time Span Repellency at a Dose of 330 µg/cm^2^

**Figure 4:**
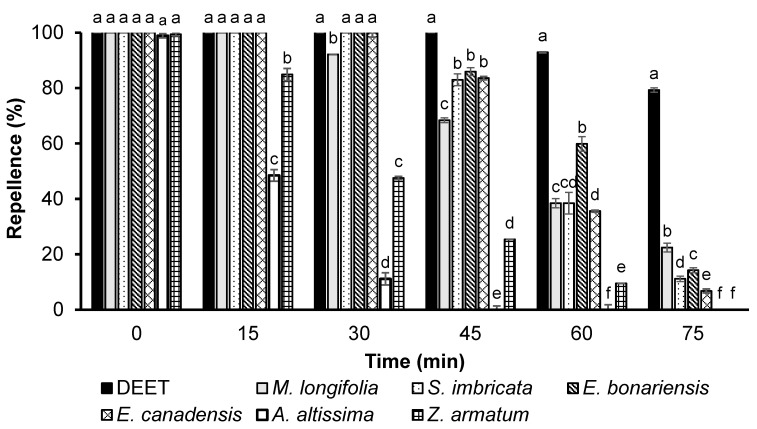
Time-based repellency of DEET and essential oils at a dose of 330 µg/cm^2^ against *Ae. aegypti*. Bars having different letters depict significant differences (*p* < 0.05) among the repellency of different tested substances at different time periods of post-treatment that were compared for each period independently by ANOVA post hoc Tukey test. Error bars denote the standard error (n = 5).

GLM analyses showed significant effects of EO type (*df *= 6, *F* = 2803, *p *< 0.001) and time (*df* = 5, *F* = 11813, *p *< 0.001) on the repellency effect. All the tested plants showed 100% repellency towards female *Ae. aegypti* when tested immediately after application, except for *A. altissima* essential oil, which showed 96.5% repellency (Figure 4). Furthermore, DEET provided complete protection for 45 min, while *E. bonariensis*, *E. canadensis*, and *S. imbricata* showed complete repellency until 30 min, against *Ae. aegypti*. However, *A. altissima* showed repellency for up to only 30 min (Figure 4). At this dose, the most active plant essential oils were *M. longifolia*, *S. imbricata*, *E. bonariensis*, and *E. canadensis*, which exhibited 70% or higher repellence for more than 45 min. After 75 min post-treatment, the repellency of DEET declined to 79%, *M. longifolia*, *S. imbricata*, *E. bonariensis*, and *E. canadensis* essential oils showed 7–22% repellency, whereas *M. longifolia* exhibited significantly higher repellence (*p* < 0.05) compared to all essential oils (Figure 4).

### 3.3. Larvicidal Activity of Essential Oils

All the tested essential oils showed larvicidal effects against the second-instar larvae of *Ae. aegypti*. There was no statistically significant difference among toxicity of *E. canadensis*, *Z. armatum*, *M. longifolia*, and *E. bonariensis* but their toxicities were significantly different from that of *S. imbricata* and *A. altissima* based on non-overlapping of fiducial limits after 24 and 48 h of post-treatment (Table 2). Furthermore, there was also a significant difference between the toxicity of *S. imbricata* and *A. altissima* based on non-overlapping of the fiducial limits after 24 and 48 h of post-treatment. The LC_50_ value of *E. bonariensis* was 28.48 mg/L after 24 h of larvae exposure which decreased to 26.03 mg/L after 48 h of post-treatment (Table 2). The tested larvae showed the least susceptibility, statistically, towards the exposure of *A. altissima* as compared to all the tested essential oils (Table 2).

### 3.4. Composition of Essential Oils

Piperitone oxide (45.5%), piperitenone oxide (30.1%), and limonene (4.6%) were the most abundant compounds in the *M. longifolia* essential oil. The major compounds in *S. imbricata* essential oil were 20% camphor, 39.9% carvone, and 6.9% piperitone, which constituted about 70% of the oil (Table 3). The *E. bonariensis* essential oil comprised *trans*-*β*-farnesene (10.2%), *cis*-lachnophyllum ester (24.9%), and matricaria ester (43.1%), whereas the major compounds of *E. canadensis* were limonene (28.4%), *cis*-lachnophyllum ester (16.3%), and matricaria ester (31.7%). The most abundant compounds in the essential oil of *A. altissima* were eugenol (24.4%) methylugenol (16.5%) and capillin (19.3%), comprising 60.2% of the essential oil (Table 3).

## 4. Discussion

Products derived from plants can be used as repellents against mosquitoes. However, their potential varies, depending upon their chemical compounds [36,37]. In the present study, essential oils of six aromatic plants, including *M. longifolia*, *S. imbricata*, *E. bonariensis*, *E. canadensis*, *A. altissima*, and *Z. armatum*, were assessed for their repellent and larvicidal effects against *Ae. aegypti*. All the essential oils showed repellency and larvicidal effects against adult females and second-instar larvae of *Ae. aegypti*, respectively. The essential oils which showed strong repellency at a dose of 33.3 µg/cm^2^ were further investigated for their longevity at tested at doses of 33.3 µg/cm^2^, 166.5 µg/cm^2^, and 330 µg/cm^2^.

The essential oil of *M. longifolia* showed the highest repellency at the lowest tested dose; moreover, these samples showed the most prolonged activity in the time span repellency bioassay. A previous study reported the repellent effect of *M. longifolia* essential oil for 65 min against *Culex pipiens* at a tested dose of 1 µL/cm^2^ (approx. 1000 µg/cm^2^), where the major compounds were 74.9% pulegone, 6.6% menthone, and 7.4% 1-8-cineole [38]. A previous study from Pakistan reported 68% repellent activity of *M. longifolia* against *Sitophilus oryzae* [39]. *M. longifolia* has also proven very effective against *Sitophilus zeamais* and showed 100% repellency [40]. Motazedian et al. [41] demonstrated that the essential oil of *M. longifolia* possessed killing and repellent ability against *Tetranychus urticae*. Koc et al. [42] reported the repellent effect (73.8%) of *M. longifolia* against *Ochlerotatus caspius*. The study of Saeidi and Moharramipour [43] also demonstrated the repellence activity of *M. longifolia* against *Tribolium confusum*.

In our study, 45.5% piperitone oxide and 30% piperitenone oxide were the major components of the *M. longifolia* essential oil and possibly contributedtowards the higher repellency of this EO against *Ae. aegypti*. Furthermore, the lower volatility of these compounds could be the reason behind the long-lasting repellency. Previously, essential oils with trans-piperitone oxide have shown toxic effects against *Cx. pipiens* [44]. In previous studies, piperitenone oxide has been proven as an excellent repellent against *Anopheles stephensi* [45] and *Ae. albopictus* [46]. Though the repellency of piperitone oxide against *Ae. albopictus* was moderate, its combined effect was significant in the case of essential oil, which contained 23% piperitone oxide and 41% piperitenone oxide [46]. A study from India reported the presence of 32.4% piperitone oxide and 41.5% piperitenone oxide in *Plectranthus incanus* essential oil that showed excellent repellency against *Anopheles stephensi* and *Culex fatigans* [47]. Thus, the synergetic effects of different components of *M. longifiolia* essential oil make it a potent repellent for *Ae. aegypti*.

The essential oil of *E. bonariensis* did not show good activity at the lowest tested dose. However, it showed 100% repellency against the tested population of mosquitoes at higher doses, such as 166.5 µg/cm^2^ and 333 µg/cm^2^. Matricaria ester, *cis*-lachnophyllum ester, and *trans*-β-farnesene were the most abundant compounds in the essential oil of *E. bonariensis*. The presence of these major compounds along with others could be the reason for prolonged repellency at the higher concentrations. Previously, matricaria ester has shown lethal effects on *Heliothis virescens* moths [48]. The presence of matricaria ester might contribute towards the repellency of *E. bonariensis* against *Ae. aegypti*.

The essential oil of *E. canadensis* showed excellent repellency at the tested doses of 166.5 µg/cm^2^ and 333 µg/cm^2^. Interestingly, in our previous study, this plant’s essential oil showed about 85% repellence at 33 µg/cm^2^ [29], whereas in the present study the essential oil of this plant species showed about 42% repellence at a similar dose. The difference in bioactivity could be attributed to the chemistry of the essential oils, as the plant samples from each study were collected from different locations. In the current study, *cis*-lachnophyllum ester (16.3%), limonene (28.4%), and matricaria ester (31.7%) were the most abundant compounds in the essential oils of *E. canadensis,* whereas Azeem et al. [29] reported results of 41.3% limonene, 10.3% of each of germacrene D and matricaria ester, and 6.5% *cis*-lachnophyllum ester. From the comparison of both studies, it could be concluded that plants growing on different soil types could have different chemistries and hence, varied bioactivity. The essential oil of *E. canadensis* also showed strong larvicidal potential against *Ae. aegypti* having LC_50_ of 35.75 mg/L. Another study from Vietnam described that *E. canadensis* essential oil possessed strong insecticidal activity against three different species of mosquitoes including *Ae. aegypti* (LC_50_ 9.80 mg/L) and *Ae. albopictus* (LC_50_ = 18.0 mg/L), indicating the toxic effect of *E. canadensis* [49]. The difference in LC_50_ values against *Ae. aegypti* in the previous and current studies might be due to a difference in the chemical composition of *E. canadensis*.

The essential oil of *Z. armatum* showed low-to-moderate repellency against adult *Ae. aegypti*. Previously, essential oil from *Z. armatum* leaves containing *α*-pinene and linalool as major compounds depicted strong repellency against *Plodia interpunctella* [50]. Additionally, *Z. armatum* EO with a binary mixture of some other essential oils also showed good repellency against stable fly, *Stomoxys calcitrans*. Furthermore, major components of *Z. armatum * EO, including cumin aldehyde, cuminyl alcohol, limonene, and methyl cinnamate showed 82%, 74%, 74%, and 64% repellency for 30 min, respectively, against stable fly [51]. In another study, *Z. armatum* EO consisting of sylvestrene, monomethyl cinnamate, 2-tridecanone, *E*-caryophyllene, vinyl decanoate, phytol, caryophyllene oxide has shown strong toxic effects against mosquitoes [52].

Essential oils of *S. imbricata* showed good-to-excellent repellency against *Ae. aegypti* at each tested concentration. Carvone was the most abundant compound (39.9%) of *S. imbricata*. Previously, carvone has shown repellence activity against *Hylobius abiet* is [53] and *Arion lusitanicus* [54]. Camphor was the second most abundant constituent (20%) of *S. imbricata * EO, which has previously shown repellency activity, ranging from 80–100% against beetles *Sitophilus granarius*, *S. zeamais*, *Tribolium castaneum*, and *Prostephanus truncates* [55,56]. The presence of these compounds, along with other compounds, might contribute towards the repellence activity of *S. imbricata* against *Ae. aegypti*.

*A. altissima* showed quite good repellency against *Ae. aegypti* at higher tested doses, albeit for a shorter period. A previous study from China has also demonstrated the repellent effects of *A. altissima* against four stored grain pests: *Tribolium castaneum*, *Oryzaephilus surinamensis*, *Sitophilus oryzae*, and *Liposcelis paeta* [57]. *A. altissima* showed insecticidal properties against *Sitophilus* zeamaise [58]. The high volatility and absence of pungent smell in the components of *A. altissima* might contribute towards repellency for short period against *Ae. aegypti*. Furthermore, in the present study eugenole (24.4%), capillin (19.3%), and methyleugenole (16.5%) were the major constituents of *A. altissima*, while in a previous study the main constituents of *A. altissima* were apocarotenoids (17.2%), oxygenated sesquiterpenes (42.1%) caryophyllene oxide (22.7%) [59]. In another study, the main compounds of *A. altissima* were α-curcumene, α-gurjunene, γ-cadinene, α-humulene β-caryophyllene, caryophyllene oxide, and germacrene D [60]. The change in a major chemical compound of *A. altissima* in the present study and previous studies might be due to a change in the location of plants of *A. altissima*.

In the larvicidal bioassays, the LC_50_ results depicted the second-instar larvae of *Ae. aegypti* to be more sensitive to the essential oils of *E. bonariensis*, *M. longifolia*, *E. canadensis*, and *Z. armatum*, as compared to those of *S. imbricata* and *A. altissima*. The LC_50_ value for *E. bonariensis* was 28.28 mg/L and 26.03 mg/L after 24 h and 48 h exposure, respectively. The presence of major compounds, such as matricaria ester and *cis*-lachnophyllum ester with high toxicity, might be contribute to the highest larvicidal activity, but the effects of other major and minor compounds cannot be ruled out. In a previous study from Vietnam, the *Conyza* (Syn: *Erigeron*) *bonariensis* essential oil exhibited LC_50_ values of 69.71 mg/L and 63.85 mg/L after 24 h and 48 h exposure, respectively [49], results which differ from the data presented here. The reason for this clear difference could be due to the difference in the chemical composition reported in the two different studies.

The LC_50_ value of *E. canadensis* was 35.7 mg/L after 48 h, which demonstrated good larvicidal potential against *Ae. aegypti*. Both *E. bonariensis* and *E. canadensis* consisted of a similar ratio of major compounds, for example, matricaria ester and *cis*-lachnophyllum ester. However, there was the one exception of limonene that was present in abundance only in *E. canadensis*. The slight difference in their bioactivity could be attributed to the difference in this chemical composition. Hoi et al. [49] reported that the LC_50_ of *E. canadensis* essential oil and pure limonene against *Ae. aegypti* was 6.09 mg/L and 17.43 mg/L, respectively. The larvicidal activity reported by Hoi et al. [49] is higher than that we found in the current study. The difference in bioactivity could be explained based on differences in the chemistry of the essential oils as well as differences in the mosquito populations. Another previous study demonstrated that *E. canadensis* essential oil exhibited quite good LD_50_ of 14.42 mg/10 g rice against adult *T. castaneum* [28]. The relative proportions of limonene, determined in the studies carried out by Azeem et al. [28] and Hoi et al. [49], were similar.

The LC_50_ values for *S. imbricata* and *A. altissima* were 124.2 and 333.6 mg/L, respectively. In a previous study, the essential oil of *A. altissima* proved toxic against aphids, having an LC_50_ of 340.06 µg/cm [61]. However, it only showed good toxic effects against *C. quinquefasciatus*, and *Ae. aegypti* at the higher concentrations of extracts, like at 75 and 100% [62]. In our study, it showed rather good toxicity towards mosquitoes, as compared to the results of Wallace et al. [62]. This might be due to a change in the chemical composition of *A. altissima*.

The essential oil of *M. longifolia* showed strong larvicidal activity (LC_50_ 39.29 mg/L) against second-instar larvae, in addition to strong deterrence activity against adult female *Ae. aegypti*. The compounds in *M. longifolia* might be toxic, which might contribute to the larvicidal activity of *M. longifolia*. In a previous study, the essential oil of *M. longifoliam* having pipertenone (43.9%) as a major compound, showed insecticidal activity against *T. castaneum* (flour beetle) and *Callosobruchus maculatus* with LC_50_ of 13.05 µL/L [63]. In another study, *M. longifolia* having *trans*-piperitone epoxide and piperitenone oxide as major compounds provided a toxic effect against the larvae of *Cx. pipiens* [44].

The essential oil of *Z. armatum* showed strong larvicidal activity against *Ae. Aegypti*, having LC_50_ of 35.92 mg/L. In the previous study, *Z. armatum* (monoterpenes as major constituents) revealed insecticidal activity against three mosquito species, including *Ae. aegypti* (LC_50_ 54 mg/L), *An. stephensi* (LC_50_ 58 mg/L), and *Cx. quinquefasciatus* (LC_50_ 49 mg/L) [64]. Previously, *Z. armatum* (2-undecanone as a major compound) has shown larvicidal activity against *An. anthropophagus* (LC_50_ 36 mg/L), *An. sinensis* (LC_50_ 38.56) [62], *T. castaneum * with (LC_50_—25.64 mg/L) [65], and *Lasioderma serricorn* (LC_50_—13.3 mg/L) [66], showing toxic effects of *Z. armatum* similar to the toxic effects showed against *Ae. aegypti* in the present study. The essential oil of *S. imbricata* showed good larvicidal activity against *Ae. Aegypti*, having a LC_50_ value of 124.2 mg/L, and previously also proved toxic against aphid with LC_50_ 340 µg/cm^2^ [62], and *Cx. pipiens* with LC_50_ = 79.1 μg/mL [67]. The change in the toxic effects of *S. imbricata* against *Ae. aegypti* and *Cx. pipiens* might be due to a difference in the chemical composition in the *S. imbricata* or due to a difference in the tested species of the mosquito.

## 5. Conclusions

The essential oils extracted from *M. longifolia*, *Z. armatum*, *E. bonariensis*, and *C. canadensis* have the potential to manage *Ae. aegypti* at the larval stage. *M. longifolia*, *E. canadensis*, *E. bonariensis*, and *S. imbricata* essential oils exhibited prolonged mosquito-repellent activity against adult female *Ae. aegypti*. These essential oils could be used to develop cost-effective and efficient mosquito-repellent formulations for personal protection, without harming humans and the environment.

## Figures and Tables

**Table 1:** Plant material and yield of essential oils used in the study.

Scientific Name	Family	Part Used	Collection Coordinates	Elevation (m)	Yield (%)
* Mentha longifolia *	Lamiaceae	Leaves, stems	34°11′48.9″ N 73°14′51.7″ E	1236	0.36
* Salsola imbricata *	Amaranthaceae	Leaves, stems	30°15′59.6″ N 71°30′40.2″ E	124	0.01
* Erigeron bonariensis *	Asteraceae	Leaves, stems	30°16′13.5″ N 71°30′44.6″ E	124	0.21
* Erigeron canadensis *	Asteraceae	Leaves, stems	30°15′52.9″ N 71°30′04.0″ E	1183	0.21
* Ailanthus altissima *	Simaroubaceae	Leaves, stems	30°15′52.9″ N 71°30′04.0″ E	124	0.04
* Zanthoxylum armatum *	Rutaceae	Leaves	34°11′48.4″ N 73°15′43.0″ E	1370	0.76

**Table 2:** Toxicity of essential oils tested against second-instar larvae of *Aedes aegypti*.

Essential Oils	Exposure Time	LC_50_ (mg/L)	95% CI (mg/L)	LC_90_ (mg/L)	95% CI (mg/L)	Slope ± SE	Chi-Square (*df*)
* M. longifolia *	24 h	46.7 a	30.41–73.39	95.9a	61.87–169.2	6.04 ± 0.86	3.28 (5)
* S. imbricata *		132.3 b	83.94–208.4	294.0 b	187.8–516.75	7.83 ± 1.10	0.31 (5)
* E. bonariensis *		28.5 a	18.30–44.30	63.3a	40.87–110.3	5.37 ± 0.49	0.33 (5)
* E. canadensis *		37.9 a	23.92–59.97	84.2 a	53.53–148.7	5.83 ± 0.79	0.34 (5)
* A. altissima *		356.1 c	231.3–551.2	791.4 b	513.62–1376	9.42 ± 1.29	0.99 (5)
* Z. armatum *		46.5 a	29.99–72.95	103.3 a	66.33–183.03	6.16 ± 0.88	4.30 (5)
* M. longifolia *	48 h	39.3 a	20.6–71.6	120.0 a	67–79	2.64 ± 0.30	5.94 (5)
* S. imbricata *		124.2 b	106–145	223.9 b	185–304	5.00 ± 0.78	2.41 (5)
* E. bonariensis *		26.1 a	21–31.61	63.6 a	49–95	3.30 ± 0.40	2.41 (5)
* E. canadensis *		35.7 a	29.9–41.8	63.9 a	52–90	5.07 ± 0.90	0.64 (5)
* A. altissima *		333.6 c	161–631	909.5 c	517–7792	2.94 ± 0.40	7.20 (5)
* Z. armatum *		35.9 a	18.6–74.7	97.6 a	59–321	2.95 ± 0.30	14.0 (5)

LC_50_ and LC_90_: lethal concentrations at which a chemical(s) will kill 50% and 90% of tested individuals exposed to it, respectively; CI: confidence interval; SE: standard error, *df*: degree of freedom; different lower-case letters in the columns depict significant differences (*p* < 0.05) between the LC_50_ and LC_90_ values based on the non-overlapping of the fiducial limits for 24 h and 48 h separately.

**Table 3:** Composition (expressed as %) of plant essential oils based on total ion chromatogram of GC-MS.

RI	Compound	CAS Number	* M. longifolia *	* S. imbricata *	* E. bonariensis *	* E. canadensis *	* A. altissima *
929	α-Pinene *	80-56-8	0.4	1.1			
970	Sabinene	3387-41-5	0.3	0.6		1.1	
972	β-Pinene *	127-91-3	0.5				
990	β-Myrcene *	123-35-3	2.0	0.7	0.1	0.7	tr
1003	α-Phellandrene	99-83-2		0.2			0.1
1008	3-Carene	13466-78-9		0.3			
1026	Limonene *	138-86-3	4.6	2.2	2.1	28.4	0.5
1028	Eucalyptol *	470-82-6	1.1	2.3	0.5		1.7
1038	*cis*-β-Ocimene *	3338-55-4	0.5				
1047	*trans*-β-Ocimene *	3779-61-1	0.1		0.8	5.0	
1056	γ-Terpinene	99-85-4	tr	0.2			0.1
1086	Terpinolene	586-62-9	0.1	0.1			0.1
1099	Linalool *	78-70-6	0.3	0.2			0.3
1103	Nonanal	124-19-6		0.5			0.5
1141	Camphor *	76-22-2	0.1	20.4	tr		0.1
1163	Borneol *	507-70-0	0.2	1.4			0.1
1175	4-Terpineol	562-74-3		0.8	tr		0.1
1189	α-Terpineol	98-55-5	0.1	0.3	tr		0.1
1194	*cis*-Dihydrocarvone	3792-53-8		1.1			
1237	Pulegone *	89-82-7		4.1			
1242	Carvone *	99-49-0	tr	39.9	0.1		0.5
1252	Piperitone	89-81-6		6.9			0.1
1260	Piperitone oxide	57130-28-6	45.5				
1272	Isopiperitenone	529-01-1	0.2				
1285	Bornyl acetate	76-49-3		0.2			
1290	Piperitenone oxide #		2.6				
1293	Thymol *	89-83-8	0.2	0.2	tr		0.9
1298	2-Hydroxypiperitone	490-03-9	0.4	0.2			0.3
1312	2-Methoxy-4-vinylphenol	7786-61-0		2.1			0.2
1337	δ-Elemene	20307-84-0			0.2		
1342	α-Guaiene	3691-12-1			0.2		
1349	α-Cubebene	17699-14-8			0.1	0.2	0.2
1358	Eugenol *	97-53-0			0.2		29.9
1378	Piperitenone oxide *	35178-55-3	30.1		0.3		
1389	β-Cubebene	13744-15-5			0.2	0.2	
1391	β-Elemen	515-13-9	0.1	0.2	0.2	0.4	
1404	Methyleugenol	93-15-2			0.6		20.3
1414	p-Menthane-1,2,3-triol	22555-61-9	3.6				
1418	*trans*-β-Caryophyllene *	87-44-5	1.8	0.3	1.9	0.7	2.8
1436	*trans*-α-Bergamotene	13474-59-4			0.2	3.6	
1452	α-Humulene	6753-98-6			1.1	0.3	0.6
1458	*trans*-β-Farnesene	18794-84-8			10.2	2.5	
1476	γ-Muurolene	30021-74-0				0.2	0.2
1480	Germacrene D	23986-74-5		0.3	4.6	6.4	6.5
1485	β-Selinene	17066-67-0	3.9				0.2
1496	Capilline	520-74-1			3.7	0.6	23.7
1508	trans-α-Farnesene	502-61-4			0.9	0.3	1.3
1516	*cis*-Lachnophyllum ester	505-01-1		0.1	24.9	16.3	
1523	δ-Cadinene	483-76-1		0.4			
1526	Matricaria ester	505-02-2			43.1	31.7	
1549	Hedycaryol	21657-90-9		2.6			
1563	Nerolidol	142-50-7				0.3	0.3
1576	Spathulenol	77171-55-2		0.3	2.0	tr	0.4
1582	Caryophyllene oxide	1139-30-6		0.4			0.3
1646	α-Eudesmol	473-16-5		0.3			1.2
1654	Juniper camphor	473-04-1		3.5			
1715	Pentadecanal	2765-11-9			0.4		0.2
1817	Hexadecanal	629-80-1					1.9
	Total Identified		98.7	94.4	98.2	98.8	95.5

RI: retention index of a separated compound, which was calculated relative to the retention time of C_9_–C_26_ hydrocarbons using DB-5 gas chromatographic column, and the same parameters were applied for analyses of essential oils. CAS Chemical Abstract Service.* Identification of compounds was verified by comparing mass spectrum and retention index with those recorded from the injection of standard compounds. # CAS number of this Piperitenone oxide was not found. The data shown in table are approximate relative compositions, expressed as %, where tr stands for trace amount < 0.1%.

## Data Availability

Data presented in this study are available on request from the corresponding authors.

## References

[1] Dehghani R., Mousavi G.A., Ghasemi B., Ghasemi M., Saheb M., Mohamadi R. (2013). A survey on residential areas infestation to house pests (Arthropods) in Kashan. Sci. Inf. Database.

[2] WHO. Dengue and severe dengue. https://www.who.int/news-room/fact-sheets/detail/dengue-and-severe-dengue..

[3] Silva N.M., Santos N.C., Martins I.C. (2020). Dengue and Zika viruses: Epidemiological history, potential therapies, and promising vaccines. Trop Med. Infect. Dis..

[4] Stanaway J.D., Shepard D.S., Undurraga E.A., Halasa Y.A., Coffeng L.E., Brady O.J., Hay S.I., Bedi N., Bensenor I.M., Castañeda-Orjuela C.A. (2016). The global burden of dengue: An analysis from the global burden of disease study 2013. Lancet Infect. Dis..

[5] Khan J., Anwar F., Shah S.S., Qamar Z., Ullah W., Ali A., Hussain M., Ali I., Ali F., Ullah F. (2021). Dengue virus epidemics: A recent report of 2018 from district Swat, Khyber-Pakhtunkhwa Pakistan. Int. J. Mosquito Res..

[6] Imran M., Hamid Y., Mazher A., Ahmad S.R. (2021). Geo-spatially modelling dengue epidemics in urban cities: A case study of Lahore, Pakistan. Geocarto Int..

[7] Wharton-Smith A., Green J., Loh E.C., Gorrie A., Omar S.F.S., Bacchus L., Lum L.C.S. (2019). Using clinical practice guidelines to manage dengue: A qualitative study in a Malaysian hospital. BMC Infect. Dis..

[8] Al Zahrani M.R., Gharsan F.N., Al-Ghamd K.M., Mahyoub J.A., Alghamdi T.S. (2020). Toxicity of two groups of pesticides against the mosquito *Aedes aegypti*. GSC GSC Biol. Pharm Sci..

[9] Junkum A., Intirach J., Chansang A., Champakaew D., Chaithong U., Jitpakdi A., Riyong D., Somboon P., Pitasawat B. (2021). Enhancement of temephos and deltamethrin toxicity by *Petroselinum crispum* oil and its main constituents against *Aedes aegypti* (Diptera: Culicidae). J. Med. Entomol..

[10] Devine G.J., Vazquez-Prokopec G.M., Bibiano-Marín W., Pavia-Ruz N., Che-Mendoza A., Medina-Barreiro A., Villegas J., Gonzalez-Olvera G., Dunbar M.W., Ong O. (2021). The entomological impact of passive metofluthrin emanators against indoor *Aedes aegypti*: A randomized field trial. PLoS Negl. Trop Dis..

[11] Samal R.R., Kumar S. (2021). Cuticular thickening associated with insecticide resistance in dengue vector, *Aedes aegypti* L. Int. J. Trop. Insect Sci..

[12] Dos Santos C.R., de Melo Rodovalho C., Jablonka W., Martins A.J., Lima J.B.P., dos Santos Dias L., da Silva Neto M.A.C., Atella G.C. (2020). Insecticide resistance, fitness and susceptibility to Zika infection of an interbred *Aedes aegypti* population from Rio de Janeiro, Brazil. Parasit Vectors.

[13] Dos Santos E., Cohren S., Costa R. (2020). Epidemiological profile of diseases caused by *Aedes aegypti* in sanitary districts of São Luis, Brazil. Eur. J. Public Health.

[14] Fernando H.S.D., Saavedra-Rodriguez K., Perera R., Black W.C., De Silva B.N.K. (2020). Resistance to commonly used insecticides and underlying mechanisms of resistance in *Aedes aegypti* (L.) from Sri Lanka. Parasit Vectors.

[15] Rahman R.U., Cosme L.V., Costa M.M., Carrara L., Lima J.B.P., Martins A.J.J.P.n.t.d. (2021). Insecticide resistance and genetic structure of *Aedes aegypti* populations from Rio de Janeiro State, Brazil. PLoS Neglec. Tropic. Dis..

[16] Zhao Q., De Laender F., Van den Brink P.J. (2020). Community composition modifies direct and indirect effects of pesticides in freshwater food webs. Sci. Total Environ..

[17] Ali S., Ullah M.I., Sajjad A., Shakeel Q., Hussain A. (2021). Environmental and health effects of pesticide residues. Sustainable Agriculture.

[18] Uwaifo F., John-Ohimai F. (2020). Dangers of organophosphate pesticide exposure to human health. Matrix Sci. Med..

[19] Pratiwi M.A.M. (2021). The repellent activity test of rosemary leaf (Rosmarinus officinalis L) essential oil gel preparations influence on Aedes aegypti mosquito. Proceedings of Journal of Physics: Conference Series.

[20] Afify A., Potter C. (2020). Insect repellents mediate species-specific olfactory behaviours in mosquitoes. Malar. J..

[21] Qiu H., Jun H.W., Dzimianski M., McCall J. (1997). Reduced transdermal absorption of N, N-diethyl-m-toluamide from a new topical insect repellent formulation. Pharm. Dev. Technol..

[22] Calafat A.M., Baker S.E., Wong L.-Y., Bishop A.M., Morales-A P., Valentin-Blasini L. (2016). Novel exposure biomarkers of N, N-diethyl-m-toluamide (DEET): Data from the 2007–2010 National Health and Nutrition Examination Survey. Environ. Int..

[23] Oftadeh M., Sendi J.J., Ebadollahi A., Setzer W.N., Krutmuang P. (2021). Mulberry protection through flowering-stage essential oil of *Artemisia annua* against the lesser mulberry pyralid, *Glyphodes pyloalis* Walker. Food Addit. Contam..

[24] Wong C., Crystal K., Coats J. (2020). Three molecules found in rosemary or nutmeg essential oils repel ticks (*Dermacentor variabilis*) more effectively than DEET in a no-human assay. Pest. Manage. Sci..

[25] Rizvi S.A.H., Ling S., Zeng X. (2020). *Seriphidium brevifolium* essential oil: A novel alternative to synthetic insecticides against the dengue vector *Aedes albopictus*. Environ. Sci Pollut Res. Int..

[26] Stappen I., Wanner J., Tabanca N., Bernier U.R., Kendra P.E. (2021). Blue Tansy Essential Oil: *Chemical Composition*, Repellent Activity Against Aedes aegypti and Attractant Activity for *Ceratitis capitata*. Nat. Prod.Commun..

[27] Tripathi A.K., Upadhyay S., Bhuiyan M., Bhattacharya (2009). A review on prospects of essential oils as biopesticide in insect-pest management. J. Pharm. Phytother..

[28] Azeem M., Zaman T., Abbasi A.M., Abid M., Mozūratis R., Alwahibi M.S., Elshikh M.S. (2022). Pesticidal potential of some wild plant essential oils against grain pests *Tribolium castaneum* (Herbst, 1797) and *Aspergillus flavus* (Link, 1809). Arab J. Chem..

[29] Azeem M., Zaman T., Tahir M., Haris A., Iqbal Z., Binyameen M., Nazir A., Shad S.A., Majeed S., Mozūraitis R. (2019). Chemical composition and repellent activity of native plants essential oils against dengue mosquito, *Aedes aegypti*. Ind Crops Prod..

[30] Johnson H. (1937). Notes on the continuous rearing of *Aedes aegypti* in the laboratory. Public Health Rep..

[31] Zheng M.-L., Zhang D.-J., Damiens D.D., Lees R.S., Gilles J.R. (2015). Standard operating procedures for standardized mass rearing of the dengue and chikungunya vectors *Aedes aegypti* and *Aedes albopictus* (Diptera: Culicidae)-II-Egg storage and hatching. Parasit Vectors..

[32] Morlan H.B., Hayes R.O., Schoof H.F. (1963). Methods for mass rearing of *Aedes aegypti* (L.). Public Health Rep..

[33] Ali A., Wang Y.-H., Khan I.A. (2015). Larvicidal and biting deterrent activity of essential oils of *Curcuma longa*, ar-turmerone, and curcuminoids against *Aedes aegypti* and *Anopheles quadrimaculatus* (Culicidae: Diptera). J. Med. Entomol..

[34] Abbott W.S. (1925). A method of computing the effectiveness of an insecticide. J. Med. Entomol..

[35] Litchfield J.j., Wilcoxon F. (1949). A simplified method of evaluating dose-effect experiments. J. Pharmacol. Exp. Ther..

[36] Hassan E.M., El Gendy A.E.-N.G., Abd-ElGawad A.M., Elshamy A.I., Farag M.A., Alamery S.F., Omer E.A. (2021). Comparative chemical profiles of the essential oils from different varieties of *Psidium guajava* L. Molecules.

[37] Nezhadasad Aghbash B., Dehghan G., Movafeghi A., Talebpour A.H., Pouresmaeil M., Maggi F., Sabzi Nojadeh M. (2021). Chemical compositions and biological activity of essential oils from four populations of *Satureja macrantha* CA Mey. J. Essent. Oil. Res..

[38] Al-Sarar A. (2014). Chemical Composition, Adulticidal and Repellent Activity of Essential Oils From *Mentha longifolia* L. and *Lavandula dentata* L. against *Culex pipiens* L. Plant. Prot. Pathol..

[39] Saljoqi A.U.R., Afridi M.K., Khan S.A. (2006). Effects of six plant extracts on rice weevil *Sitophilus oryzae* L. in the stored wheat grains. J. Agric. Biol Sci..

[40] Odeyemi O., Masika P., Afolayan A. (2008). Insecticidal activities of essential oil from the leaves of *Mentha longifolia* L. subsp. capensis against *Sitophilus zeamais* (Motschulsky)(Coleoptera: Curculionidae). Afr. Entomol..

[41] Motazedian N., Ravan S., Bandani A. (2012). Toxicity and repellency effects of three essential oils against T *etranychus urticae* Koch (Acari: Tetranychidae). J. Agric. Sci Technol. Health Care..

[42] Koc S., Oz E., Cetin H. (2012). Repellent activities of some Labiatae plant essential oils against the saltmarsh mosquito *Ochlerotatus caspius* (Pallas, 1771)(Diptera: Culicidae). Parasitol Res..

[43] Saeidi M., Moharramipour S. (2013). Insecticidal and repellent activities of *Artemisia khorassanica*, *Rosmarinus officinalis* and *Mentha longifolia* essential oils on *Tribolium confusum*. J. Crop. Prot..

[44] Koliopoulos G., Pitarokili D., Kioulos E., Michaelakis A., Tzakou O. (2010). Chemical composition and larvicidal evaluation of *Mentha*, *Salvia,* and *Melissa* essential oils against the West Nile virus mosquito *Culex pipiens*. Parasitol. Res..

[45] Tripathi A.K., Prajapati V., Ahmad A., Aggarwal K.K., Khanuja S.P. (2004). Piperitenone oxide as toxic, repellent, and reproduction retardant toward malarial vector *Anopheles stephensi* (Diptera: Anophelinae). J. Med. Entomol..

[46] Giatropoulos A., Kimbaris A., Michaelakis A., Papachristos D.P., Polissiou M.G., Emmanouel N. (2018). Chemical composition and assessment of larvicidal and repellent capacity of 14 Lamiaceae essential oils against *Aedes albopictus*. Parasitol. Res..

[47] Pal M., Kumar A., Tewari K.S. (2011). Chemical composition and mosquito repellent activity of the essential oil of *Plectranthus incanus* link. Facta Univ. Ser. Phys. Chem. Technol..

[48] Binder R.G., Chan B.G., Elliger C.A. (1979). Antibiotic Effects of C10–C12 Fatty Acid Esters on Pink Bollworm, Bollworm and Tobacco Budworm. Agric. Biol. Chem..

[49] Hoi T.M., Huong L.T., Chinh H.V., Hau D.V., Satyal P., Tai T.A., Dai D.N., Hung N.H., Hien V.T., Setzer W.N. (2020). Essential oil compositions of three invasive *Conyza* species collected in Vietnam and their larvicidal activities against *Aedes aegypti*, *Aedes albopictus*, and *Culex quinquefasciatus*. Molecules.

[50] Brari J., Kumar V. (2021). Insecticidal efficacy of essential oils from *Artemisia maritima* L. and *Zanthoxylum armatum* DC. and their two major constituents against *Plodia interpunctella* (Hubner). Int. J. Life Sci. Res..

[51] Hieu T.T., Kim S.I., Kwon H.W., Ahn Y.J.J.P.m.s. (2010). Enhanced repellency of binary mixtures of *Zanthoxylum piperitum* pericarp steam distillate or *Zanthoxylum armatum* seed oil constituents and *Calophyllum inophyllum* nut oil and their aerosols to *Stomoxys calcitrans*. Pest. Manage. Sci..

[52] Singh A., Dhami A., Palariya D., Prakash O., Kumar R., Kumar R., Pant A. (2019). Methyl nonyl ketone and linalool rich essential oils from three accessions of *Zanthoxylum armatum* (DC.) and their biological activities. Int. J. Herb. Med..

[53] Schlyter F., Smitt O., Sjödin K., Högberg H.E., Löfqvist J. (2004). Carvone and less volatile analogues as repellent and deterrent antifeedants against the pine weevil, *Hylobius abietis*. J. Appl. Entomol..

[54] Frank T., Biert K., Speiser B. (2002). Feeding deterrent effect of carvone, a compound from caraway seeds, on the slug *Arion lusitanicus*. Ann. Appl. Biol..

[55] Obeng-Ofori D., Reichmuth C., Bekele A., Hassanali A. (1998). Toxicity and protectant potential of camphor, a major component of essential oil of *Ocimum kilimandscharicum*, against four stored product beetles. Int. J. Pest. Manag..

[56] Chen Z.-Y., Guo S.-S., Cao J.-Q., Pang X., Geng Z.-F., Wang Y., Zhang Z., Du S.-S. (2018). Insecticidal and repellent activity of essential oil from *Amomum villosum* Lour. and its main compounds against two stored-product insects. Int. J. Food Prop..

[57] Lu J., Wu S. (2010). Bioactivity of essential oil from Ailanthus altissima bark against 4 major stored-grain insects. Afr. J. Microbiol. Res..

[58] Haroon M., Maduka U., Sujarajini V. (2018). Insecticidal effects of plant extracts of some medicinal plants against *Sitophilus zeamaise* mostchulsky on stored maize. Abstracts of the 7th Annual Science Research Sessions (ASRS).

[59] El Ayeb-Zakhama A., Ben Salem S., Sakka-Rouis L., Flamini G., Ben Jannet H., Harzallah-Skhiri F. (2014). Chemical composition and phytotoxic effects of essential oils obtained from Ailanthus altissima (Mill.) swingle cultivated in Tunisia. Chem. Biodivers.

[60] Kozuharova E., Benbassat N., Berkov S., Ionkova I. (2020). *Ailanthus altissima* and *Amorpha fruticosa*–invasive arboreal alien plants as cheap sources of valuable essential oils. Pharmacia.

[61] Khani A., Ordouni F., Sahebzadeh N.J.E.a.B. (2018). Qualitative phytochemical screening and mortality effect of ethanolic extract of Salsola imbricata on *Aphis gossypii*. Exp. Anim. Biol..

[62] Wallace J.R., Wylie C.D., Wagner R.L. (2021). Plant extract efficacy on mosquito mortality: Preliminary studies on the effect of Ailanthus altissima extract on adult *Aedes aegypti* and *Culex quinquefasciatus*. Great Lakes Entomol..

[63] Khani A., Asghari J. (2012). Insecticide activity of essential oils of *Mentha longifolia*, *Pulicaria gnaphalodes* and *Achillea wilhelmsii* against two stored product pests, the flour beetle, *Tribolium castaneum*, and the cowpea weevil, *Callosobruchus maculatus*. J. Insect Sci..

[64] Tiwary M., Naik S., Tewary D.K., Mittal P., Yadav S.J.J.o.v.b.d. (2007). Chemical composition and larvicidal activities of the essential oil of *Zanthoxylum armatum* DC (Rutaceae) against three mosquito vectors. J. Vector. Borne Dis..

[65] Zhang W., Wang Y., Geng Z., Guo S., Cao J., Zhang Z., Pang X., Chen Z., Du S., Deng Z. (2018). Antifeedant activities of lignans from stem bark of *Zanthoxylum armatum* DC. against *Tribolium castaneum*. Molecules.

[66] Wang C.-F., Zhang W.-J., You C.-X., Guo S.-S., Geng Z.-F., Fan L., Du S.-S., Deng Z.-W., Wang Y.-Y. (2015). Insecticidal constituents of essential oil derived from *Zanthoxylum armatum* against two stored-product insects. J. Oleo Sci..

[67] Abutaha N., Al-Mekhlafi F.A., Al-Keridis L.A., Farooq M., Nasr F.A., Al-Wadaan M. (2018). Larvicidal potency of selected xerophytic plant extracts on *Culex pipiens* (Diptera: Culicidae). Entomol. Res..

